# Improving Questions on Sexual Partnerships: Lessons Learned from Cognitive Interviews for Britain’s Third National Survey of Sexual Attitudes and Lifestyles (“Natsal-3”)

**DOI:** 10.1007/s10508-012-9962-2

**Published:** 2012-06-14

**Authors:** Catherine R. H. Aicken, Michelle Gray, Soazig Clifton, Clare Tanton, Nigel Field, Pam Sonnenberg, Anne M. Johnson, Catherine H. Mercer

**Affiliations:** 1 Centre for Sexual Health and HIV Research, Research Department of Infection and Population Health, University College London, 3rd Floor Mortimer Market Centre, off Capper Street, London, WC1E 6JB UK; 2 National Centre for Social Research, London, UK

**Keywords:** Epidemiology, Sexual partnerships, Sexual mixing, Cognitive interview, Sex surveys

## Abstract

Patterns of sexual partnership formation and dissolution are key drivers of sexually transmitted infection transmission. Sexual behavior survey participants may be unable or unwilling to report accurate details about their sexual partners, limiting the potential to capture information on sexual mixing and timing of partnerships. We examined how questions were interpreted, including recall strategies and judgments made in selecting responses, to inform development of a module on recent sexual partnerships in Britain’s third National Survey of Sexual Attitudes and Lifestyles (“Natsal-3”). Face-to-face cognitive interviews were conducted with 14 men and 18 women aged 18–74 years, during development work for Natsal-3. People with multiple recent partners were purposively sampled and questions were presented as a computer-assisted self-interview. Participants were generally agreeable to answering questions about their sexual partners and practices. Interpretation of questions designed to measure concurrent (overlapping) partnerships was broadly consistent with the epidemiological concept of concurrency. Partners’ ages, genders, ethnicity, and participants’ perceptions of whether partner(s) had had concurrent partnerships were reported without offense. Recall problems and lack of knowledge were reported by some participants (of all ages), especially about former, casual, and/or new partnerships, and some reported guessing partners’ ages and dates of sex. Generally, participants were able to answer questions about their sexual partners accurately, even when repeated for multiple partners. Cognitive interviews provided insight into the participants’ understanding of, ability to answer, and willingness to answer questions. This enabled us to improve questions used in previous surveys, refine new questions, and ensure the questionnaire order was logical for participants.

## Introduction

### The Importance of Understanding Sexual Partnerships and Sexual Mixing

The prevalence of sexually transmitted infections (STI) has been shown to vary by gender, age, and ethnic group (Aral, 2000; Fenton et al., 2001; Garnett et al., 1996; The UK Collaborative Group for HIV & STI Surveillance, 2007). STI transmission is determined by numbers and characteristics of sexual partnerships. Risk increases with increasing partner numbers, but is also related to partner’s STI risk and protected or unprotected sex (Anderson, May, Boily, Garnett, & Rowley, 1991; Aral, 2000; Aral et al., 1999; Fenton et al., 2001; Garnett et al., 1996). STI transmission risk varies according to whether partnerships are formed between people from similar (“assortative”) or different (“disassortative”) prevalence and sexual activity groups (Garnett et al., 1996). For example, young women with older male partners are at increased risk, relative to young women who form partnerships with men of a similar age (DiClemente et al., 2002; Ford, Sohn, & Lepkowski, 2001; Miller, Clark, & Moore, 1997).

In addition to the characteristics of sexual partners and partnerships, their timing is important. Concurrent partnerships are those in which an individual has two or more partnerships which overlap in time, as opposed to “serial monogamy” where one partnership ends before another one starts. Concurrency has the effect of placing all those in the partnership network at risk and can be thought of as effectively increasing the network of partners, because a larger number of people are at risk if an STI is introduced. The proportion of partnerships which are concurrent has an impact on transmission at the population level (Morris & Kretzschmar, 1995; Watts & May, 1992).

Understanding the characteristics of sexual partners and partnerships in the general population is therefore important for our understanding of STI transmission dynamics and can inform appropriate targeting of interventions. However, while concurrency is a particularly important parameter to measure for STI epidemiology, measurement is problematic because of the difficulties of remembering accurately and the social undesirability of reporting such partnerships in the context of a societal norm against “unfaithfulness.”

### Challenges of Collecting Data on Sexual Partnerships Through Surveys

The collection of sexual behavior survey data is recognized as challenging for a variety of reasons (Catania, Gibson, Chitwood, & Coates, 1990; Meston, Heiman, Trapnell, & Paulhus, 1998; Wight & West, 1999). The need for detailed data on sexual partners’ characteristics and the number and timing of sexual partnerships adds to this challenge, for several reasons.^1^

First, the highest burden is often placed on a subgroup of particular interest. Participants with multiple recent partners may be asked questions repeatedly, for each partner, but typically up to a certain number (e.g., their two or three most recent partners) (Juarez & Martin, 2006; Kraut-Becher & Aral, 2006; Luke, 2005), increasing interview duration and, potentially, recall difficulties. Researchers must balance the epidemiological benefit of collecting detailed information against the risks of over-burdening some participants and jeopardizing the validity of the data. Participant fatigue or irritation may decrease accuracy of responses, introduce non-response or even cause the interview to be terminated. Bias may be introduced where these risks relate to particular participant characteristics (including particular partnership histories).

Second, the questions involve participants thinking about their partnerships with specific individuals (which may have since ended), which may be more emotionally sensitive than thinking about their sexual behavior in general.

Third, while ability to recall information about sexual behavior can depend on the salience, recency, and social significance of the experience, this may vary between participants’ partnerships. Participants may be asked for information which they may have never known (a partner’s exact age, for example).

Fourth, in order to avoid asking directly about “affairs” or non-monogamy, which can be an especially sensitive topic, measures of concurrency can be derived indirectly, and potentially inaccurately, based on approximate partnership dates.

Finally, gathering these data from general population samples means that questions and response options must be appropriate not only to participants with a wide range of sexual behaviors and experiences, but also to a range of sexual partnerships, from brief, anonymous sexual encounters to very long, committed relationships. Much of the recall error and bias in survey data may be attributable to aspects of questionnaire design (as well as interviewing methods) (Friedenreich, 1994); hence, measures taken to improve design will increase the accuracy and comparability of data to be collected about varied partnerships, from varied participants.

The British National Surveys of Sexual Attitudes and Lifestyles (Natsal) are the largest probability surveys of sexual behavior undertaken anywhere in the world to date. Two surveys have been undertaken, a decade apart, and this article describes part of the development work for the third survey, Natsal-3. Natsal-1 and Natsal-2 sampled the age groups 16–59 and 16–44, respectively, while Natsal-3 seeks to sample 16–74 year olds. A key objective of probability surveys such as Natsal (Fenton et al., 2001; Johnson et al., 2001; Johnson, Wadsworth, Wellings, & Field, 1994; Wellings et al., 2001; Wellings, Field, Johnson, Wadsworth, & Bradshaw, 1994) is to provide data on sexual partnerships and sexual mixing in the general population. We sought to address the methodological issues discussed above in the development of Natsal-3, specifically its “most recent partnerships” (MRP) module. This module included new partnership-specific questions as well as questions asked in previous Natsal studies (Erens et al., 2001; Wellings et al., 1994).

We used cognitive interviewing to test the new and revised questions and the overall flow of this module and to learn how people understand concepts such as concurrency. Cognitive interviews explored the ways in which participants understood survey questions and formulate responses, and this study took place prior to a pilot in which the survey procedures and survey would be tested as a whole.

## Method

### Participants

A total of 32 cognitive interviews were conducted. The cognitive interviews were administered in two consecutive phases in 2008–2009, with different targeted recruitment strategies. Phase A involved interviews with 22 participants aged 16–74 years who had taken part in the National Centre for Social Research (NatCen) Omnibus survey in February–March 2008 and indicated their willingness to assist with further studies. For practical reasons concerning the areas the interviewers working on this study could cover, participants were interviewed in Scotland, North East England, North West England, and Yorkshire.

Participants were recruited into quotas which ensured a gender and age balance reflective of the expected Natsal survey population; no further selection criteria were employed for Phase A. Phase B comprised interviews with ten participants recruited via an email advertisement sent out to NatCen staff. The request to pass the advert onto friends and family aimed to seek people who had had more than one sexual partner in the last 5 years, in order to test the module with people more likely to find its repetitive nature burdensome. Naturally, some Omnibus participants (Phase A) also met this criterion.

The purposive sampling strategy was designed to reflect the diversity of the proposed survey population, covering characteristics anticipated to influence reactions and response to the questions, including: gender (9 men and 13 women in Phase A, 5 and 5 respectively in Phase B); age (in Phase A, 13 aged 16–58 and 9 aged 59–74 years; in Phase B, 10 in the younger age range); and number of recent sexual partners (in Phase A, 2 out of 22 Phase A participants reported two or more partners in the last 5 years; in Phase B, all 10 participants reported this). In terms of participant’s *own* concurrency, only one participant in Phase B reported this and so answered this question. All ten Phase B participants answered the question about *partner*’*s* concurrency. Neither of these questions was asked in Phase A.

### Measures and Procedure

**Fig. 1:**
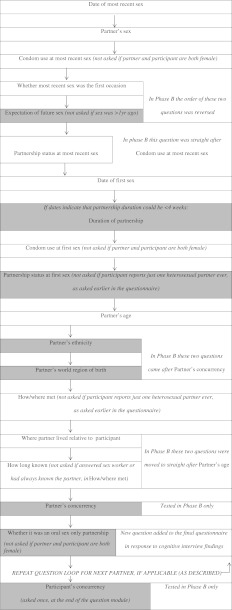
Most recent partner “loop” showing question order. *Shaded boxes* were new questions, *clear boxes* existing questions (which in some cases had been modified from Natsal-2). Questions were specific to each sexual partner/partnership, so for “first sex” read “first sex with this partner.” Question order in the final questionnaire was as in Phase B, with one additional question (whether the sexual partnership was oral sex only, i.e., not vaginal or anal sex), and the question on partner’s world region of birth removed

As in previous Natsal studies, all sexually experienced participants in Natsal-3 were asked questions about their most recent partner’s characteristics, regardless of how long ago this partnership occurred. If participants reported two or more partners in the 5 years prior to the survey interview, the question loop repeated for their second and third most recent partners. If, earlier in the questionnaire, a male participant reported sexual partners of both genders, but only reported details of female partners among his ≤3 most recent partners in the 5 years prior to interview, the question loop was repeated for his most recent male partner (and vice versa for female participants). Figure 1 shows the MRP question loop and the wording of questions and responses are provided in the Appendix Table 1.

**Table 1:** Question wording

Question topic	Initial question wording (all questions were partner-specific)	Initial response options	Phase in which the question was tested	Final wording and response options
Partner’s initials or nickname (used as an aide-memoire)	(*If more than one partner in the last 5* *years*:) To make it easier to remember the answers to these questions, please type in a nickname or the initials of the person you had sex with most recently/your second most recent partner/your third most recent partner. This is just to help you remember who you are answering the questions about so it does not have to be their real name. The name or initials you type in will not be used in any way and will be deleted from the laptop at the end of the questionnaire	(*participant enters a nickname/initial*)	B	(*If more than one partner in the last 5* *years*:) To make it easier to remember the answers to these questions, please type in a nickname or an initial for the person you had sex with most recently/second most recent person you had sex with/third most recent person you had sex with. This is just to help you remember who you are answering the questions about, so a made up nickname or initial is fine. No one will see this nickname or initial except you and it will be deleted from the laptop at the end of the questionnaire
Date of most recent sex	When was the most recent occasion you had sex with that person? (*Instruction added in phase B*:) Please estimate if you can’t say exactly	(*year, month*—*including option for* ‘*can*’*t remember the month*’ (*Phase A*)*, later changed to* ‘*I am unable to estimate*’ (*Phase B*)*. Month is not asked if* >*5* *years ago*)	A, B^a^	*Instruction wording altered*: If not sure of the exact month or year please give your best estimate *Question wording and response options unchanged from Phase B*
Partner’s sex	Is that person female or male? (*Or*:) Is that person male or female?	Male Female	A, B^a^	*Unchanged*
Condom use at most recent sex	(*Female participants*:) Was a condom used on that most recent occasion? (*Male participants*:) Did you use a condom on that most recent occasion?	Yes No	A, B	*Additional instruction*: If you had only oral sex, and not vaginal or anal sex, on this most recent occasion, please choose answer option 3 (“*we only had oral sex on the most recent occasion*”), even if you did use a condom *Additional response option*: We only had oral sex on the most recent occasion
Whether most recent sex was the first occasion	Was that (most recent) occasion also the FIRST occasion with that person, or not?	Yes, the first occasion No, not the first occasion	A, B^a^	*Question wording unchanged* *Response options*: Yes—I have only had sex with (*him/her*) once No—I have had sex with (*him/her*) on more than one occasion
Expectation of future sex	Do you think you will have sex with this person again in the future? (*Not asked where no sex in previous year*)	Yes Probably Probably not No I don’t know	A, B^a^	Are you likely to have sex with this person^a^ again in the future? *Response options unchanged*
Partnership status at most recent sex	Which one of these descriptions applies best to you and that person at the time you MOST RECENTLY had sex?	We were married/in a civil partnership/living together as a couple at the time We were in a steady relationship at the time We were not in a steady relationship at the time	A, B^a^	*Wording of the main question unchanged. Sub*-*question removed* *Response options altered*: We were living together as a couple/married/in a civil partnership at the time We were in a steady relationship at the time We used to be in a steady relationship, but were not at that time We had known each other for a while, but were not in a steady relationship We had recently met We had just met for the first time
	(*and if not in a steady relationship*) You mentioned that you were **not** in a steady relationship with this person. Which one of these best applies to you and this person at the time you most recently had sex with them?	We used to be in a steady relationship, but were not at that time We had known each other for a while, but were not in a steady relationship We had recently met We had just met for the first time	A, B^a^	
Date of first sex	When was the FIRST occasion with that person?	(*year, month*—*including option for* “*can*’*t remember the month*”*.* (*Phase A*)*, later changed to* “*I am unable to estimate*” (*Phase B*)*. Month is not asked if* >*5* *years ago*)	A, B^a^	*Unchanged from Phase B*
Duration of partnership	*Only asked if first sex was same or previous calendar month to the interview. Wording depends on response to expectation of future sex*: (*if yes or probably*) How long ago was it that you first had sex with this person? (*If no or probably not or don*’*t know*) How long was it between the first and last time you had sex with this person?	Less than 7 days Between 7 days and 2 weeks Between 2 and 4 weeks Over 4 weeks	A, B^a^	*Unchanged*
Condom use at first sex	(Female participants:) And was a condom used on that first occasion with that person? (Male participants:) And did you use a condom on that first occasion with that person?	Yes No	A, B^a^	*Additional instruction*: If you had only oral sex, and not vaginal or anal sex, on this most recent occasion, please choose answer option 3 (“*we only had oral sex on the most recent occasion*”), even if you did use a condom *Additional response option*: We only had oral sex on the most recent occasion
Partnership status at first sex	Which one of these descriptions applies best to you and that person at the time you FIRST had sex?	We were married/in a civil partnership/living together as a couple at the time We were in a steady relationship at the time We were not in a steady relationship at the time	A, B^a^	*Wording of the main question unchanged. Sub*-*question removed* *Response options altered*: We were living together as a couple/married/in a civil partnership at the time We were in a steady relationship at the time We used to be in a steady relationship, but were not at that time We had known each other for a while, but were not in a steady relationship We had recently met We had just met for the first time
	You said you were **not** in a steady relationship with that person. Which one of these descriptions best applies to you and that person at the time you FIRST had sex?	We used to be in a steady relationship, but were not at that time We had known each other for a while, but were not in a steady relationship We had met recently We had just met for the first time	A, B^a^	
Partner’s age	How old was that person on the FIRST occasion you had sex together? (*Instruction added in phase B*:) Please estimate if you can’t say exactly	(*age in years, option for* “*I don*’*t know*”*, later changed to* “*I am unable to estimate*” *in Phase B*)	A, B^a^	How old was that person^a^ on the FIRST occasion/when you had sex together? *Response options unchanged from Phase B*
Partner’s ethnicity	Which ethnic group does that person belong to?	White or White British Mixed ethnicity Asian or Asian British Black or Black British Chinese or other ethnic group Don’t know	A, B^a^	Which ethnic group or background does (or did) that person^a^ belong to?
				*Response options unchanged*
	(*If Black*:) What is his/her cultural background?	Caribbean African Other Black background Not sure	A, B	What is (or was) that person’s^a^ background? *Response options unchanged*
Partner’s world region of birth	Was this person born in the UK? (*In Phase B*: Do you know which country (*name*) was born in?)	Yes No Not sure	A, B^a^	*Removed from final questionnaire*
	(*If no*) Which part of the world was he/she born in?	(*In Phase B response options included* “*The UK*”) Other European countries (including Ireland, Eastern Europe, Russia) Australia, New Zealand North America (USA and Canada) South America, Central America (including Mexico) Caribbean countries Asian countries (including China, India, Pakistan, Bangladesh, Thailand, Malaysia, etc.) Middle East, North Africa African countries (other than North Africa) Other region or country (*participant can enter free text*) Don’t know which region or country	A, B	*Removed from final questionnaire*
How/where met	Where did you FIRST meet that person?	At school At university or college At work (or through work) In a pub, bar, night club or disco Introduced by friends or family Through a sports club, faith group, or other organization or society On holiday or while travelling Internet dating website Other dating agency/personal ads Chat room, social networking site or online gaming Had always known each other (for example as family friends or neighbors) Neighbor/lived locally/house or flatshare Arranged marriage In a public place (e.g., park, museum, shop, public transport) (*He/she*) was a sex worker/prostitute Other (*if this is selected, participant can then enter free text*)	A, B^a^	How did you FIRST meet that person^a^? *Minor changes* (*underlined*) *to these three response options*: In a pub, bar, nightclub, dance, or disco Online, but not through a dating website(*replaces* Chat room, social networking site or online gaming) Through an arranged marriage
Where the partner lived relative to the participant	When you FIRST met that person, where did (*he/she*) normally live?	In the same town or city as you did In the same region as you, but in a different town In a different region, but the same country as you In a different country from you Don’t know	A, B^a^	*Unchanged*
How long known	How long had you known this person before you first had sex? (*not asked if responded* “*sex worker*” *or* “*always known each other*” *to the question how/where met*)	24 h or less Between 1 day and 1 week Between 1 and 4 weeks Between 4 weeks and 6 months Between 6 months and 1 year Between 1 and 5 years Between 5 and 10 years 10 years or more	A, B^a^	How long was it between when you first met that person^a^ and when you first had sex with (*him/her*)? *Further information could be viewed on request, as follows*: This question is asking about the length of time from when you **first met** this person to when you first had sex with (*him/her*), not the length of time from when you first entered into a relationship There may have been a gap between first meeting them and when you got to know (*him/her*) properly, but we would still like you to count from the very first meeting (for example this could have been face-to-face, over the phone, or online) *Response options unchanged*
Partner’s concurrency	*If first sex with this partner was more than 5* *years ago and partnership is ongoing*: Do you think that (*name*) has had sex with anyone else in the last 5 years? *If first sex with this partner was more than 5* *years ago and most recent sex with this partner was in the last 5* *years and partnership is not ongoing* Do you think (*name*) had sex with anyone other than you between (*month and year 5* *years ago*) and (*month and year of last sex with this partner*) (the most recent occasion you had sex with him/her) *If first sex with partner was less than 5* *years ago and partnership is ongoing* Do you think (*name*) has had sex with anyone else since you first had sex together? *If first sex with partner was less than 5* *years ago and partnership is not ongoing* Do you think (*name*) had sex with anyone else in the time between when you first and most recently had sex together?	Yes Probably No Prefer not to say	B	*Question wording unchanged* *Response options*: Yes Probably Probably not No Prefer not to say
Oral sex only partner	–	–	–	*New question*: *For heterosexual partnerships*: Thank you for answering about this person. Can we just check, was *that person* ^*a*^ someone you had oral sex with, but never vaginal or anal sex? *For homosexual male partnerships*: Thank you for answering about this person. Can we just check, was *that person* ^*a*^ someone you had oral sex with, but never anal sex? *No corresponding question for homosexual female partnerships* *Response options*: Yes No
Participant’s concurrency	*Where concurrency not clear from dates of partnerships*: Just to check, was there any overlap between (*name A*) and (*name B*)? In other words, was the first time you had sex with (*name A*) before the last time you had sex with (*name B*)?	Yes—there was overlap No Not sure Prefer not to say	B	Thank you for answering those questions about the people you have had sex with most recently. Just to check, was there any overlap between (*name A*) and (*name B*)? In other words, was the first time you had sex with (*name A*) before the last time you had sex with (*name B*)? *Response options unchanged*

^a^Wording changed from “this/that person” to the nickname entered at the start of each partner loop, in phase B, and also in the final questionnaire among participants who completed the questionnaire about more than one most recent partner. It remains “this person” or “that person” in the final questionnaire where participants are answering about only one partner

The cognitive interviews took place in participants’ homes or researchers’ offices and were audio-recorded. After giving informed consent, participants completed a selection of questions from the Natsal-3 draft survey (including the entire draft MRP module and other questions not reported here). In order to test the questions as they would be administered in the “real” survey, and because routing between questions was complex, cognitive interviews used the same method of administration for the questions as planned for Natsal-3, i.e., computer-assisted self-interview (CASI) for the entire MRP module. To help ensure privacy during completion, interviewers did not look at the CASI responses at any point. Periodically, participants were instructed to stop completing the questions and the interviewer asked about the preceding questions.

The main cognitive interviewing techniques were *probing* (the interviewer asked specific questions to gain an understanding of how the participant went about answering the questions) and *think aloud* (the participant was asked to voice their thoughts as they completed the questionnaire) (Collins, 2003). At the end of the MRP module (as with other groups of questions not reported here), cognitive interviewers used *retrospective* probing, for which some probes were pre-scripted. Interviewers showed the questions on flash-cards in order to remind participants to which question they were referring. As probing tends to be less burdensome on participants than think aloud (Collins, 2003), and for reasons of confidentiality, interviewers did not ask participants to think aloud as they completed the CASI (although if participants spontaneously told the interviewer what they were thinking this was not discouraged).

Examples of the cognitive interview probes included: (1) “What do you think this question is getting at?” (2) “How did you feel about being asked this question?” (3) “How easy or difficult did you find this question to answer?” (4) “Were you able to find a suitable answer option, or do you think there were any options missing?” (5) “Were you able to provide [the requested information] or did you guess?” (6) “How accurate would you say your guess was?” In addition, and specific to the MRP module, interviewers asked about the question order and flow, as one aim was to test whether the question order was logical for participants. Through these methods, cognitive interviews assessed: (1) acceptability; (2) whether questions were understood as intended; (3) understanding of key concepts, including concurrency; (4) ability to recall the information sought (including recall strategies and judgments made in formulating answers) and to provide an answer. Interviews lasted 1–1.5 h each (including sections of the questionnaire not reported here) and participants were given a £20 voucher as a token of appreciation.

Structured notes were made by the interviewer upon completion of each interview. These were analyzed using Framework, which allows a case-and-theme-based structure to be derived (Ritchie, Spencer, & O’Connor, 2003). A matrix was created, listing the questions across the page, and cases (participants and brief demographic characteristics) down the page. Under each question, researchers summarized how the participant understood the question, recall strategies used, judgments made in formulating an answer, any problems, and the answer itself. Therefore, data could be read as case records for each participant, or question by question, across all cases.

Ethical approval for this study was provided by NatCen’s Research Ethics Committee, ref i9699.

## Results

### Overview and Flow of Questions

Participants found the loop of questions about their most recent partners (Fig. 1, Appendix Table 1) somewhat taxing, but for the most part logical and straightforward. Some participants who completed the question loop more than once commented that they would have liked to know how many times the partner loop would be repeated, because the burden of recall that this might involve was distracting for them.

### Using a Nickname to Identify Partners

At the beginning of the question loops, the questionnaire instructed participants to enter a name, initials or a nickname for each partner, which would be deleted at the end of the module. This was a new addition to the Natsal-3 survey, intended to help participants focus on each partner in turn and was tested in Phase B (it was not tested in Phase A, where the majority of participants were answering about just one partner).

Most participants were comfortable with this and reported that it was helpful for recall. However, some participants had not realized that they could invent a name (and did not wish to enter a partner’s real name; in one case, a participant found it an unpleasant reminder that she had not known the name of a sexual partner). Another participant requested additional confirmation that the names would not be used by researchers. It was important to emphasize that names could be made up and would be deleted, as after this reassurance was given, all Phase B participants were comfortable entering names, nicknames or initials.

The word “partner” appeared in the introduction to each question loop (except the first) and caused some confusion. Due to its social significance, this word was not used elsewhere in the module, where partners were referred to as “the person you had sex with” or by nickname/initials.

### Sexual Partners’ Characteristics

Participants were asked each partner’s age at the time when they first had sex with each other, partner’s ethnicity, and where the partner was born (country or world region). Although partners’ ages had been asked in previous Natsal surveys (the other two items were new), participants spontaneously expressed difficulty in answering this accurately for some partners.

Questions on partners’ ethnicity and region of birth aimed to provide epidemiologically important data, since prevalence of STIs differs by ethnicity and country of birth (Health Protection Agency Centre for Infections, 2008). Broad response categories were provided and, if participants selected “Black”’ or “Black British,” they were asked a further question, reflecting differences in the prevalence of HIV and other STIs between people of Black Caribbean and Black African origin in the UK (Health Protection Agency Centre for Infections, 2008). Questions about partners’ ethnicity did not offend or upset participants and were readily answered for brief and longer-term partnerships (informed guesses were made, usually based on appearance, if participants were unsure). One participant commented that the ethnic background of her partners did not matter to her and queried the purpose of this question.

In contrast, it was much more difficult for participants who had not known their partners for long to answer the question about partners’ country/world region of birth. In addition, participants queried the purpose of the question: one participant, for example, commented “*Why should I know, and why would I want to know where that person was born?*” (referring to partners she did not know very well). Some participants who did not know the answer reported that it made them feel “*guilty*” and as if they were being judged for not knowing this information. In Phase A, participants were asked whether each partner was born in the UK and if not, they were asked to choose the region of birth from a list. In Phase B, the wording was changed, intending to avoid the suggestion that participants ought to know the answer: participants were asked whether they knew the partner’s country of birth and, if so, they were asked to choose from a list of world regions including the UK (see Appendix Table 1). Despite wording changes, the question was still perceived by some as “*out of the blue*” and “*strange*” (and the same participant who had queried the purpose of the ethnicity question, asked why country of birth was asked in addition to ethnicity)*.*

### How Participants Met Their Partners

The question about how participants first met each partner listed 16 response options, including the new option “chat room, social networking site or online gaming” (Appendix Table 1). The question was tested to explore whether response options were comprehensive. The question was clear and easy to understand, with participants consistently understanding the term “met” as when they first spoke to each other or had some kind of social exchange. Some minor queries were raised, such as what to do if multiple categories applied e.g., “university” and “at a social event.” Despite the length of the list (which was commented upon), participants reported no difficulty in selecting a response.

No participants chose “chat room, social networking site or online gaming,” so participants in Phase B were specifically asked what they thought it meant. Participants seemed to look at the three examples in this response separately, rather than considering it as an overall “online” category (excluding online dating, which was listed separately). Some of the meanings participants attached to “online gaming” were not necessarily as intended, with participants universally thinking of online poker rather than other gaming sites such as “Second Life” or “World of Warcraft.” However, it should be noted that as they had not reported meeting partners this way, participants were being asked to examine this category somewhat artificially.

### How Long Participants Had Known Their Partner When They First Had Sex with Them

This question was clear and participants generally understood it as intended, counting from the time when they first met until when they first had sex. Some participants reported using life or employment circumstances to assist recall. In one case, a participant counted from the first date with a partner whom she had known for longer, while another female participant admitted tending to round up from her initial estimation, in order to select a response, “*my inner prude coming out.*” Perceived accuracy varied between participants and partnerships and the time ranges provided were considered helpful.

### Partnership Dates

Ability to recall dates depended on the recency and salience of the experience, among participants of all ages. Not surprisingly, months were more difficult to recall than years. Recall strategies included thinking about the season and other life events. In Phase A, participants were given a “can’t remember” response option, yet were able to recall accurately the month and year of most recent sex, particularly when it was very recent. As Phase B included participants who had multiple recent partners, the wording was adjusted, asking participants to “estimate the month if you can’t say exactly,” and this was found to be helpful.

### Reporting Partnership Status at Most Recent Sex

Participants were asked their relationship status with each partner when they most recently had sex with each other. This was a new question for Natsal-3 (Appendix Table 1).

One response option grouped marital, civil, and cohabiting partnerships together (“we were married/in a civil partnership/living together as a couple at the time”) and occasionally the cohabiting part was overlooked. Seeing the term “married,” some participants who were cohabiting with a partner selected “we were in a steady relationship […]” instead.

The term “steady relationship” was received in different ways: some found it appropriate, compared with alternatives, but others found it “*weighted*” or unfamiliar (“*it sounded American*”). Meanings participants attached to “steady relationship” covered the following themes: monogamy, length of relationship, social recognition of the relationship, frequency of seeing each other, purpose, and “*whether it*’*s going somewhere,*” all broadly in line with the information sought by researchers. Participants also proposed alternatives including “long-term relationship,” “committed relationship,” or just “relationship.” The term “steady relationship,” though not particularly popular, was intuitively and consistently understood. Problems distinguishing between “steady” and “not steady” relationships appeared to be due to partnerships being in transition between the two states, rather than comprehension. Where partnerships were in the early stages, “*on the rocks*” or ongoing but casual and non-committed, finding a response was more difficult, while the question was easy to answer for stable relationships, including marriages.

### Reporting Likelihood of Having Sex Again with Partners

This question aimed to measure whether a partnership was ongoing and used to route later questions and to weight partnership-level data in analyses (Copas, Mercer, Farewell, Nanchahal, & Johnson, 2009; Mercer et al., 2009). It was not asked when no sex was reported in the previous year. In Natsal-2, whether or not a partnership was ongoing was estimated during analysis, based on responses to three other questions. Asking a direct question is likely to be more accurate than making such assumptions and was found to be easy to answer and inoffensive; some participants who were married or in relationships found it humorous. The only minor issue concerned the distinction between a participant’s desire—*wanting* to have sex with a partner again—and the perceived *likelihood* of this happening, with one participant commenting that he would have liked to answer “*hopefully!*” Between Phase A and Phase B, a “don’t know” category was added and this was found to be helpful.

### Reporting Own Concurrency and Perceptions of Partners’ Concurrency

These questions, developed while Phase A was in the field, were tested in Phase B. Own concurrency (i.e., a participant having overlapping sexual partnerships) was assessed from partnership dates (month and year), with an extra question to check whether there was any overlap where this could not be ascertained from the dates provided. Just one participant met these criteria and was therefore routed to this question. This person understood the question well and reported no problems.

Questions about each partner’s concurrency (i.e., whether the partner had any other sexual partners during their sexual partnership with the participant) focused on the last 5 years, to match with the period upon which the module focused, to aid recall and to standardize the question across partnerships of different lengths. There were four versions of this question for each combination of: partnerships beginning within the last 5 years or before this time; and partnerships which have ended or were ongoing (see Appendix Table 1 for question wording). It was difficult to explore each version in turn as participants were asked different versions for different partners and it was difficult to distinguish between the different question versions during probing. However, no participants reported finding the question confusing, so it is likely that its most complex version did not cause major comprehension issues.

As anticipated, participants generally found partners’ concurrency an uncomfortable topic. Reassuringly, however, even participants who reported that their partners had had concurrent partnerships were not offended by these questions.

Regarding accuracy, while some participants considered their perception of a partner’s concurrency as “*factual*” and their response very accurate, participants who did not know for certain still considered their answers reasonably accurate. We acknowledge that the accuracy of these data will be questionable, as it is impossible to know for sure that a partner did *not* have sex with others. The research team considered that there was no real difference between “probably not” and “no,” yet participants preferred a graded scale (i.e., the addition of “probably not”).

Participants were asked what they thought these questions were trying to ascertain, and answers (including: “*going behind your [partner*’*s] back,*” “*cheating on someone,*” “*having an affair*”) were generally consistent with the epidemiological concept of concurrency (UNAIDS Reference Group on Estimates Modelling, and Projections: Working Group on Measuring Concurrent Sexual Partnerships, 2010). However, no participants interpreted the question as including sex with another partner while “on a break” from a relationship, although this scenario is important for STI transmission and thus important to measure.

### Definitions of Sex

In Natsal-3, at the start of the CASI, participants were given a definition of sex, inclusive of vaginal, oral, and anal intercourse. To explore whether participants were using this definition throughout the MRP module, after completing the module, participants were asked to write down their own definitions and in Phase B they were asked to indicate whether they were thinking of vaginal, oral, and anal sex when they answered the MRP questions. Although participants understood the definition provided, this was sometimes at odds with their own definitions, which did not always include oral sex, stating for example: “*I don*’*t see [oral sex] as* ‘*sex*’” and “*for me the definition of sex is vaginal intercourse.*” In the cognitive interviews, some participants revealed that they reverted to their own definitions when answering questions about their most recent partners, leaving out partners with whom they only had oral sex.

## Discussion

### Statement of Principal Findings

Our cognitive interview findings suggest that survey participants sampled from the British general population are likely to be able to answer many questions about their recent sexual partners, providing detailed data to inform analyses of sexual partnerships and sexual mixing in this population. Even highly sensitive questions about sexual partners, such as those on concurrency, were generally acceptable and did not cause offense among people of different age or gender, or among those who had had multiple recent partners. Our findings also demonstrated the potential of cognitive interviewing to explore aspects beyond question wording, such as the use of nicknames to assist participants in answering about multiple partners. While most participants had just one or a few partners during the recall period, the diversity of sexual behavior required that a balance be struck between asking about a limited number of partners and asking about all partners in a specified time period. Our findings suggest that, in this context, it is likely to be feasible and acceptable to ask detailed questions about up to three or four recent partners.

### Strengths and Weaknesses

Cognitive testing allowed refinement of existing questions and testing of new questions and the MRP module as a whole. This process helped to address potential problems, before testing the survey and survey procedures as a whole in the larger-scale pilot. Our research contributes to a small but growing literature on the use of cognitive interviews in sex survey research (e.g., Deardorff, Tschann, & Flores, 2008; Edwards, Thomsen, & Toroitich-Ruto, 2005; Macdonald et al., 2008; Mavhu, Langhaug, Manyonga, Power, & Cowan, 2008; McCabe, Tanner, & Heiman, 2010), and as far as we are aware it was the first to focus on questions about sexual partners and partnerships.

Certain questions were reached through routing from other questions, applied to certain partners only, and therefore were tested on a subset of participants. We would expect major problems with a question to be revealed even with a few participants. However, problems specific to certain unusual participant or partner/partnership characteristics are less likely to surface. That said, several questions had been successfully used before, and the sampling was designed to reach people with a range of characteristics, particularly older age groups which were not surveyed in previous Natsal studies and those with multiple recent partners.

Although we concluded that participants did not find the questions offensive, the full version of the module (including questions about own and partners’ concurrency) was only asked of participants in Phase B, who were younger (aged under 40), and had responded to an advertisement seeking people who had had more than one sexual partner in the last 5 years. This purposive sampling strategy sought to test the questions among varied participants and those who would be completing the question loop repeatedly (rather than to be representative of Natsal participants). We may have missed problems the questions posed for other groups of people. Cognitive interview participants may also have been particularly willing to assist with research or particularly comfortable with revealing this type of sensitive information, compared to the eventual Natsal-3 survey participants. As with any pre-testing, it should not be assumed that participants in the main survey will react in the same way. Indeed, cognitive interviews to pre-test survey questions are carried out not to replace but to complement a pilot, in which some assessment of the response among a larger and possibly more “representative” sample of participants can take place. However, it is reassuring that the subsequent pilot study has supported the conclusions of the cognitive interviews in terms of the feasibility of asking these questions and their acceptability to participants (Phelps, Ogunbadejo, & Nicholson, 2009).

Targeting participants who had had multiple recent partners (Phase B) proved a successful way of testing the questions for a variety of partnership types (for instance, different durations and from casual to cohabiting partnerships), each participant describing at least two partnerships. However, while we succeeded in sampling participants with a range of characteristics, sampling participants whose partner(s)/partnership(s) had particular characteristics would have been challenging, because we could not have used the questions which were being tested in the recruitment process. This led to some limitations: most partners were reported to be white, for example, and so the question on partners’ ethnicity was not tested extensively among people who had partners of different ethnicities.

### Meaning of the Study

Participants were generally happy to answer questions about sexual partners’ demographics and none were perceived as too intrusive. However, when participants did not know certain details about a particular partner, the feeling of being “caught out,” as if judged as not really knowing this person, was uncomfortable for some participants who had not known their partners for long. This was a particular problem for the question on partner’s country/region of birth and, compounding this problem, the purpose of the question was not clear to participants. For these reasons, and because it seemed likely to be answered inaccurately or not at all for newer and casual partnerships, this question was removed from the Natsal-3 questionnaire.

Ordinarily, sex survey researchers seek information about behavior and participant characteristics, with the implicit assumption that participants normally know, or at some previous point knew, the information necessary to respond. Questions about participants’ partners may seek information never known to the participant. Researchers need to be aware of this; although information requested in a question may not seem sensitive, lack of knowledge about a partner can be sensitive in itself. In the case of partner concurrency, there were particular concerns about validity. However, we were reassured that participants considered their responses to be accurate and retained this question due to its epidemiological importance.

Further changes were made to the Natsal-3 questionnaire in order to reduce frustration and item non-response, where cognitive interview participants indicated difficulties in giving an accurate response. For instance, wording was changed to instruct participants to “*estimate if you can*’*t say exactly*” the timing of events within the 5 years prior to interview and their partner’s age when they first had sex with them. Reducing the amount of missing data for the latter variable was important, as in Natsal-2 14 % of non-regular partners’ ages were missing. Further minor changes were made to question wording and order.

Where few problems with a question were reported, no changes were made if it was judged that while a change could improve a question for some participants, it could cause problems for others. For example, the term “steady relationship,” though unpopular, was understood consistently and alternatives were considered equally or more ambiguous.

In some cases, potential improvements might result in loss of comparability with previous Natsal surveys, so although informed by the cognitive interview findings, the final decisions were referred back to the study team. For example, repeatedly emphasizing the Natsal definition of sex (by providing a definitions card or presenting the definition at the start of the module) in Natsal-3 might introduce bias, if participants to Natsal-1 and Natsal-2 were discounting partners with whom they had only had oral sex.

### Conclusions

Through testing the whole MRP module, our findings revealed problems with questions which had been used in previous Natsal surveys that had not been identified. This demonstrates the potential of cognitive interviewing to reveal problems not identified by expert panels or through previous use of questions in the field suggesting that cognitive testing of questions which have already been field-tested remains valuable in improving question wording and in ascertaining whether topics are acceptable, understood and can be answered with reasonable accuracy. It also supports the approach Natsal-3 development work took in using cognitive pre-testing to complement a conventional pilot.

## Appendix

See Table 1.

## Footnotes

1 Throughout this article, we use the terms “partner” and “partnership” to refer to any sexual partner or partnership, irrespective of its duration or social significance.
